# Identification of Ifitm1 as a Pivotal Gene in Mouse Spinal Cord Injury Using Comprehensive Machine Learning Algorithms

**DOI:** 10.1155/mi/6149780

**Published:** 2025-08-11

**Authors:** Yu Yao, Kun Xi, Shaohu Xu, Feiyu Zhang, Liang Chen

**Affiliations:** ^1^Department of Orthopedics, The First Affiliated Hospital of Soochow University, Orthopedic Institute, Soochow University, Suzhou, Jiangsu, China; ^2^Department of Spine Surgery, Affiliated Hospital of Nantong University, Nantong, Jiangsu, China

**Keywords:** hdWGCNA, Ifitm1, machine learning algorithms, single-cell data analysis, spinal cord injury

## Abstract

**Background:** Spinal cord injury (SCI) functions as a medical condition leading to substantial motor and neurological system deteriorations. Researchers must understand the molecular mechanisms of this disease because it serves as a foundation for creating therapeutic solutions.

**Methods:** This study analyzed the single-cell dataset GSE189070 and microarray datasets GSE47681, GSE92657, and GSE93561 retrieved from the GEO database. Using R packages “Seurat” and “Celldex,” we identified and annotated cell clusters in single-cell data. Combined microarray datasets underwent differential expression analysis, WGCNA, and machine learning to identify key hub genes. Immune cell associations were assessed using Cibersort, while the connection map (CMap) database was employed to predict small-molecule drugs targeting the identified genes. Experimental validation confirmed findings.

**Results:** In datasets involving single cells, granulocyte subpopulations denote unique cellular populations related to SCI. The high-dimensional weighted gene co-expression network analysis (hdWGCNA) algorithm pinpointed crucial modules linked to granulocyte subgroups, particularly from the black, green, and yellow modules. In the SCI cohort, Ifitm1 emerged as a potential hub gene. Importantly, Ifitm1 shows a significant positive correlation with M1 macrophages. Utilizing the CMap database along with molecular docking investigations, the small-molecule drug NVP-AUY922, which interacts with Ifitm1, was discovered. Experimental assessments revealed that Ifitm1 is linked to macrophage inflammation following SCI.

**Conclusion:** This study revealed the importance of granulocyte subsets and Ifitm1 in SCI, proposed Ifitm1 as a potential therapeutic target, and provided new insights into the molecular mechanisms of SCI.

## 1. Introduction

Spinal cord injury (SCI) denotes harmful alterations that occur following a sudden contusion or a break in the anatomical continuity of the spinal cord, which may result from either direct or indirect trauma [1]. This condition is complex and highly destructive, frequently triggered by traumatic incidents. SCI can lead to significant and lasting neurological and motor impairments, ultimately causing enduring disability that profoundly affects the patient's quality of life and treatment possibilities [2, 3]. Every year, hundreds of thousands of people worldwide suffer from spinal cord injuries, which pose significant economic and psychological pressures on healthcare systems and affected families due to their high disability rates, treatment costs, and long-term care needs [4]. The main causes of SCI are diverse, including mechanical injury, surgical complications, tumors, infections, and other related factors [5–8]. Importantly, traumatic events account for over 90% of SCI occurrences, stemming from scenarios, such as road accidents, acts of violence, sports injuries, or falls. The process that leads to SCI includes both primary and secondary forms of injury. The term primary injury denotes the mechanical harm done to the spinal cord, where spinal cord transection interferes with impulse transmission, resulting in either temporary or ongoing dysfunction of sensory, motor, and autonomic nervous system functions. Such injury can swiftly trigger a range of intricate pathophysiological alterations, such as bleeding, edema, ischemia, and cellular apoptosis [9]. The death of cells following an injury, along with the activation of inflammatory cells in affected areas of neural tissue, may lead to further damage. This additional injury encompasses intricate pathophysiological processes, such as imbalances in ions, oxidative stress due to free radicals, inflammatory reactions, and the development of glial scars [10, 11]. Primary SCI is unavoidable and permanent; consequently, existing therapeutic approaches mainly focus on reducing the following pathological alterations caused by secondary injury, thus improving the outlook for individuals affected by SCI [12]. Although more and more studies have extensively explored SCI, there have been no significant breakthroughs in the treatment of SCI [13]. The key reason for this situation is that the treatment of nerve regeneration and functional recovery still faces significant challenges. Firstly, the low regenerative capacity of the central nervous system (CNS) is one of the main obstacles to SCI treatment. Secondly, the complex pathophysiological changes after SCI further increase the difficulty of treatment. In addition, the lack of targeted and effective regeneration induction strategies is also an important obstacle to the progress of SCI treatment. The current treatment methods mainly focus on reducing inflammation, inhibiting oxidative stress, and promoting axonal regeneration, but the efficacy of these strategies is still limited in clinical applications [14]. The complex molecular mechanisms and pathophysiological processes related to SCI are multifaceted, involving the interactions of multiple cell types, signaling pathways, and molecular networks. Therefore, it is crucial to thoroughly elucidate the cellular and molecular processes that drive SCI in order to develop innovative and effective therapeutic strategies.

During the pathological progression of SCI, immune cells such as granulocytes, macrophages, and lymphocytes swiftly gather at the site of injury and play a significant role in the inflammatory response. Previous research has highlighted the importance of immune cell infiltration in the context of SCI. Specifically, Wang et al. [15] and colleagues showed that CCL28 acts as a protective element after SCI by attracting CCR10-positive and immunosuppressive regulatory T cells. The signaling pathway of CCL2/CCR2 plays a vital role in the recruitment of T cells to sites of SCI, indicating its potential as an innovative therapeutic target [16]. Research conducted by Milich et al. [17] and colleagues revealed a close association between the pathogenesis of SCI and the features and behavior of macrophages derived from infiltrating monocytes. In conclusion, evaluating the infiltration of immune cells is crucial for revealing the molecular mechanisms that underlie SCI and for discovering new targets for immunotherapy [18, 19].

With the development of technology, bioinformatics plays a key role in the research of tumors and nontumor diseases, providing new ideas and technical support for understanding complex disease mechanisms, accurate diagnosis, and personalized treatment. Bioinformatics integrates and analyzes multiple omics data to reveal mutations, diverse gene expression patterns, and key signaling pathways in the tumor microenvironment, aiding in the identification of potential therapeutic targets and biomarkers [20–22]. Single-cell data analysis technology helps reveal key biological processes in various diseases by analyzing gene expression, chromatin status, and cell heterogeneity at the single-cell level [23–27]. Machine learning, as a rapidly developing and emerging field, possesses powerful capabilities for handling large, complex, and heterogeneous data. Multiple machine learning algorithms have been widely applied in the research of tumor and nontumor diseases, providing powerful tool support for key feature screening, pattern recognition, and predictive modeling [28–34]. As a result, this research utilized bioinformatics techniques, including single-cell analysis, high-dimensional weighted gene co-expression network analysis (hdWGCNA), and machine learning, to explore changes in the immune microenvironment after SCI, aiming to shed light on the variations in key immune-related molecules involved in the disorder. Additionally, we identified small-molecule drugs aimed at potential biomarkers as possible therapeutic targets. The results of this study seek to provide new insights and therapeutic approaches for the management of SCI.

## 2. Materials and Methods

### 2.1. Data Download and Processing

We obtained three microarray datasets (GSE47681, GSE92657, and GSE93561) from the GEO database. The GSE47681 dataset includes five samples from wild-type controls, 12 samples from wild-type SCI cases, four samples from mutant controls, and 13 samples from mutant SCI cases. In GSE92657, there are three samples designated as controls and three associated with SCI, whereas GSE93561 is made up of six control samples alongside six SCI samples. We will combine these three datasets into a comprehensive SCI research cohort. The “limma” package is used for data normalization. The “sva” R package is used to eliminate batch processing effects when integrating different datasets [35, 36]. The single-cell sequencing data for mouse SCI tissue utilized in this study were obtained from the GSE189070 dataset, which includes sequencing data from seven SCI samples and one normal sample.

### 2.2. Analysis of Single-Cell SCI Data in Mice

In this study, the R package “Seurat” was utilized to create Seurat objects from data gathered through single-cell RNA sequencing [37]. In the beginning, we implemented quality control measures to remove cells that had fewer than 200 expressed genes, exceeded 2500 expressed genes, or had mitochondrial gene compositions greater than 10%. We conducted principal component analysis (PCA) focusing on the top 2000 variable genes and selected 10 principal components to facilitate dimensionality reduction and clustering. The “Harmony” function was employed to synchronize the samples and reduce batch effects. Subsequently, using the results from Harmony, we applied the RunUMAP function for Unified Manifold Approximation and Projection (UMAP) dimensionality reduction, while utilizing the FindNeighbors and FindClusters functions for neighborhood assessment and clustering analysis to elucidate the data structure. Then, use the FindAllMarkers function to search for differentially expressed genes (DEGs) in each cluster. Finally, we use the “SingleR” package to annotate the type of each cell population.

### 2.3. hdWGCNA

The hdWGCNA provides a solid methodological framework for the inference, analysis, and interpretation of gene co-expression networks from high-dimensional transcriptomic data, including data obtained from single-cell and spatial transcriptomic studies [38]. In our study, we adopted the hdWGCNA technique to construct a scale-free network at the level of individual cells. The R package “hdWGCNA” was utilized to enhance network connectivity, where the threshold for the scale-free topology model was established at greater than 0.85, and a soft threshold of 9 was chosen. To assess similarities in gene expression, we generated a topological overlap matrix (TOM) using the Pearson correlation coefficient and then built a clustering dendrogram with the “PlotEndprogram” function. We classified the genes into separate modules and examined the connections between these modules and cell clusters by employing Module Eigengenes to identify the most relevant module. To pinpoint the hub genes within these modules, we selected three unique modules and retrieved the top 100 genes from each as hub genes. Functional enrichment analysis was performed on these hub genes using the “clusterProfiler” package.

### 2.4. WGCNA Analysis of SCI Queue

We employed the R package “WGCNA” to pinpoint modules that are closely linked to SCI and their associated core genes. Initially, we organized all samples and removed genes with an average expression below 0.5. To improve the identification of strong correlations among modules, we used the “pickSoftThreshold” function to find the ideal soft threshold power (*β*). Subsequently, we transformed the expression matrix into both an adjacency matrix and a TOM. We determined the dissimilarity of the TOM matrix (dissTOM) and grouped a minimum of 60 genes into distinct modules using the dynamic tree cutting algorithm. Following this, we employed the “WGCNA” software package to investigate interaction strengths, assess gene significance (GS) and module membership (MM), as well as to explore the connections between modules and clinical characteristics. Genes that displayed high GS and MM from the modules of interest were deemed key genes and selected for further analysis. Lastly, we used the “heatmap” package to depict the association between modules and clinical characteristics [39].

### 2.5. Identification of DEGs

In our analysis of SCI samples compared to normal samples, we employed the “limma” R package to perform a differential expression assessment, with the objective of identifying DEGs. We established a log fold change (logFC) threshold of 1 and set a corrected *p* value criterion of below 0.05.

### 2.6. Multiple Machine Learning Algorithms Are Used to Screen Potential Biomarkers Related to SCI

An analysis of intersection among hub genes, DEGs, and core genes highlighted through hdWGCNA will be carried out. Following this, we will apply three machine learning methods, Boruta, SVM-RFE, and random forest, to the identified intersecting genes in order to pinpoint potential biomarkers linked to SCI. Boruta proves reliable because it examines feature importance by referencing randomly selected features. Boruta effectively stops overfitting because it operates best on high-dimensional data and identifies feature interactions during the process. It determines the significance score of each feature and contrasts it with shadow features generated randomly, thereby assisting in the detection of the genes that are most pertinent to SCI [40]. In this research, we applied SVM-RFE analysis to pinpoint the most significant feature genes linked to SCI. The random forest algorithm constructs a multitude of decision trees to form a forest aimed at predicting the ultimate outcome [41, 42]. In this study, we used the “randomForest” package to identify the most relevant feature genes to SCI. However, Boruta has high computational overhead, especially when dealing with very high-dimensional data, which may consume a long time to calculate, and is sensitive to noise in the data; SVM-RFE is sensitive to hyperparameter settings, and improper parameter selection may affect the model effect, and the computational complexity is high; the interpretability of the random forest model is poor, it is difficult to clearly understand the relationship between features and prediction results, in some cases it may lead to overfitting, and it consumes a lot of computational resources. Therefore, the three methods complement each other and can be used to analyze and screen potential biomarkers from different perspectives. By employing these machine learning methodologies to extract feature genes for intersection analysis, we identified the key feature genes pertinent to SCI and delved deeper into their roles in both disease progression and potential treatments. Following this, we evaluated gene expression and the diagnostic significance of biomarkers within the SCI cohort. An independent *t*-test was implemented to analyze the expression levels of biomarkers in SCI samples compared to normal samples, with a significance threshold established at *p*  < 0.05. Furthermore, the receiver operating characteristic (ROC) curve was utilized to assess the clinical diagnostic efficacy of these biomarkers.

### 2.7. Immune Infiltration Analysis

CIBERSORT has been utilized to evaluate the prevalence of various immune cell types using gene expression data [43]. We examined the ratios of 22 unique immune cell types within the SCI cohort via the CIBERSORT algorithm. To assess the abundance of immune cells in both SCI and control samples, we conducted the Wilcoxon test. Differences in the distribution of immune cells across the SCI and control groups were visualized using the “vioplot” package. Furthermore, we explored the correlation between potential biomarkers and immune cells, representing these relationships using lollipop plots.

### 2.8. GSEA Analysis of Potential Biomarkers

The enrichment of the top-ranked genes across the pathways of these groups was evaluated using GSEA software. In this evaluation, the C2.CP.KEGG. v7.2 gene set from the MSigDB database was employed, with the number of gene sets analyzed in each instance set to 1000. Significant differences among gene sets were identified by implementing criteria that included a nominal (NOM) *p* value of less than 0.05 and a false discovery rate (FDR) *q* value below 0.25.

### 2.9. Potential Drug Prediction and Molecular Docking

The connection map (CMap) database explores the relationship between gene expression and small-molecule drugs, which assists researchers in identifying drugs that are closely associated with different diseases. In this research, we employed the CMap database to discover potential small-molecule drugs. We conducted molecular docking using the online tool CB-Dock2, followed by an extensive visual evaluation. CB-Dock2 serves as a cutting-edge blind docking tool aimed at improving docking precision. It forecasts the binding site of a specified protein by calculating the center and size through a method based on curvature-driven cavity detection and works in conjunction with the advanced docking software AutoDock Vina. Furthermore, CB-Dock2 organizes the binding modes according to Vina scores and offers interactive 3D visualizations of these binding configurations, listing the binding energy values from highest to lowest [44, 45].

### 2.10. Animals

In this research, we employed adult male C57BL/6 mice weighing between 18 and 22 g. These mice were from an experimental animal center, and the experiments were conducted in accordance with ARRIVE guidelines, with prior approval from the Nantong University Experimental Animal Center and the Nantong University Experimental Animal and Plant Protection and Use Committee (S20241101-100).

### 2.11. Mouse SCI Model

Anesthesia was commenced by inhaling sevoflurane gas (RWD, Shenzhen, China) at a concentration of 3%, which was subsequently maintained at 1%. To ensure that the body temperature remained stable at 37°C throughout the surgical intervention and recovery phase, the mice were placed on a thermostatically regulated electric blanket. Each mouse was secured, and a laminectomy was performed at the T10–T11 spinal segment to expose the dorsal side of the spinal cord while keeping the dura mater intact. A 5-gram weight was then dropped from a height of 30 mm to strike the exposed spinal cord, leading to a traumatic SCI. Following this, the muscle and skin layers were stitched using 4–0 silk sutures. Conversely, mice in the Sham group received anesthesia and laminectomy but did not experience SCI. After the SCI, the animals were injected with penicillin intramuscularly to fight infection, and then returned to clean cages for individual rearing, and the cages were changed in a timely manner to keep warm after the operation. Throughout the experimental period, animal care, including manual bladder milking, was performed twice a day to promote urination after SCI. SCI mice were euthanized, perfused, and sampled on the third day after SCI.

### 2.12. Adeno-Associated Virus (AAV) Utilization

Adult mice (6–8 weeks) were anesthetized using 1%–2% isoflurane (RWD, Shenzhen, China) inhalation and fixed on a 37°C preheated plate to dilate the tail vein. A caudal lateral vein with a diameter of approximately 0.3 mm was selected and sterilized with 75% alcohol, and then slowly punctured at an angle of 15°–30° using a 31 G insulin needle. After blood return to confirm successful entry, AAV suspension (1 × 10^11^ vg per mouse, volume = 200 μL) was injected at a uniform rate (100 μL/min). After injection, pressure was applied to stop bleeding for 1 min, iodine povidone was sterilized twice, and the mice were placed on a warm recovery pad for observation for 30 min.

### 2.13. Fluorescence-Activated Cell Sorting (FACS)

Immune cells infiltrating the injured spinal cord were evaluated by flow cytometry. Briefly, mice were dislocated, and the spinal cord was quickly removed. The spinal cord tissue was homogenized and passed through a 70 μm nylon cell filter. The homogenate is centrifuged at 600 × *g* for 5 min, and the pellet is resuspended in a 70% isotonic Percoll reservoir. Cells were separated by Percoll gradient (0%–70%) and centrifuged at 2000 × *g* for 20 min. The supernatant and myelin fraction were carefully removed. The residual percoll was then washed away, and the cell fraction was resuspended in PBS containing 2% fetal bovine serum. To differentiate the immune cell populations, mouse CD45 APC, CD11b FITC, and F4/80 PE antibodies (all from eBioscience, USA) were used. Cells were evaluated by FACS AriaTM flow cytometer (BD Biosciences, USA). The data were analyzed by FlowJo v10 software.

### 2.14. Cell Culture and Transient Transfection

RAW264.7 cells were sourced from Beijing Bena Biotechnology Co. in Beijing, China, and were cultivated in DMEM F-12 medium. The cells were transfected with siRNA targeting Ifitm1 (Sagon, China) as well as a negative control (NC) using Lipofectamine 2000 from Invitrogen, a part of Thermo Fisher, USA. The sequence of si-Ifitm1 is shown in Table 1.

### 2.15. Quantitative Reverse Transcriptase Polymerase Chain Reaction (qRT-PCR)

Total RNA was extracted from treated BV2 or PC12 cells using Trizol reagent (Life Sciences), in accordance with the manufacturer's instructions. The concentration of RNA was measured with a NanoDrop spectrophotometer (Thermo Fisher Scientific). Subsequently, RNA was converted into cDNA utilizing the HiScript II Q RT SuperMix kit (Vazyme, Cat# R122-01), which was used as a template for quantitative polymerase chain reaction (qPCR). The qRT-PCR was carried out with the AceQ-qPCR SYBR Green Master Mix (Vazyme, Cat# Q122-01) along with a real-time fluorescent quantitative PCR system (Roche). The expression levels of the target genes were calibrated against the reference gene β-actin, following the 2^-ΔΔCt^ methodology. All primers are shown in Table 2.

### 2.16. Immunofluorescence Staining

Mice's spinal cords were embedded in paraffin following fixation in 10% formalin, dehydration, and slicing into 5 μm sections. The spinal cord slices then underwent deparaffinization with xylene and were dehydrated using a gradient of alcohol. A citrate buffer facilitated antigen retrieval, and hydrogen peroxide was employed to inhibit endogenous peroxidase activity. The slices were permeabilized using 0.5% Triton X-100 for 5 min at room temperature. Subsequently, coverslips were treated with 1% BSA for 15 min at room temperature before incubation overnight at 4°C with F4/80 (Cell Signaling Technology #24595, dilution 1:50) and Ifitm1 (Abcam #ab119857, dilution 1:50). Following five washes, the cells were incubated with the relevant secondary antibodies for 1 h at room temperature. Images were captured using an Olympus Fluorview-3000 confocal microscope (Olympus Optical Corporation).

### 2.17. Neurobehavioral Assessment

The modified neurological severity score (mNSS) test was conducted on mice before the injury and again on days 1, 7, 14, and 21 after the injury. This thorough evaluation comprised assessments of reflexes, sensory perception, and motor capabilities. Scores on the test varied from 0 to 18, where elevated scores signified a greater level of functional impairment.

### 2.18. Statistical Analysis

The statistical analyses were carried out utilizing R software (version 4.3.3). A *t*-test for independent samples was used to examine the differences between the two groups, with a *p* value of below 0.05 regarded as statistically significant.

## 3. Results

### 3.1. Single-Cell RNA Sequencing Analysis Reveals Cellular Heterogeneity in Mouse SCI Tissues

**Figure 1:**
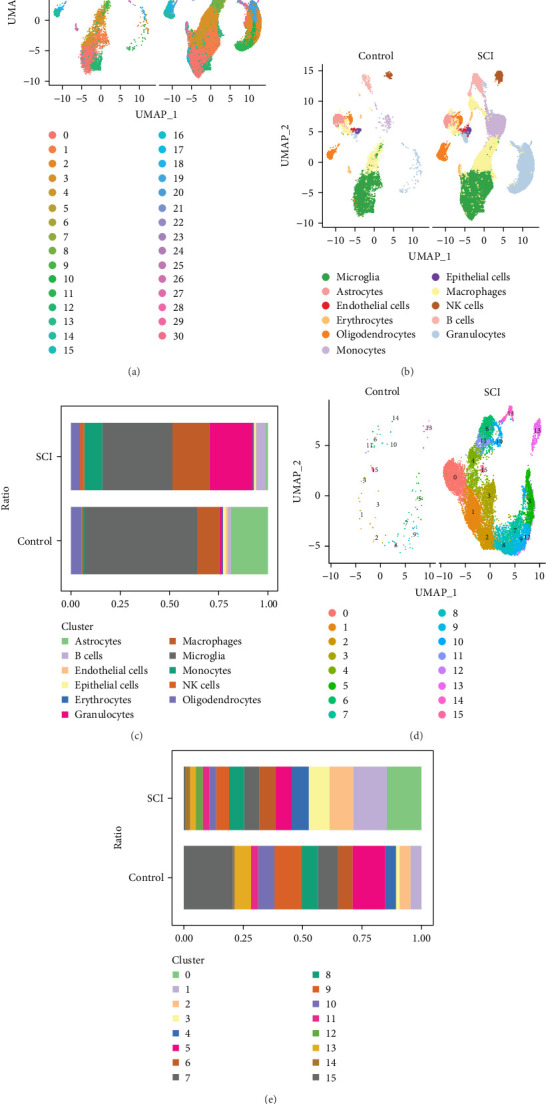
Key subpopulations of granulocytes were identified through single-cell analysis. (A) A total of 31 distinct cell clusters across all samples were revealed by UMAP analysis. (B) These 31 clusters were classified into 11 diverse cell types. (C) The comparison of the 11 cell types was conducted between the SCI sample group and the control sample group. (D) Further dimensionality reduction clustering analysis of granulocyte subgroups resulted in a UMAP plot illustrating 16 distinct cell clusters. (E) The analysis of the proportions of clusters associated with granulocyte subgroups was performed between the SCI sample group and the control sample group.

This research employed the GSE189070 dataset obtained from the GEO database. To start, stringent quality control protocols were applied, leading to the discovery of 31 cell clusters via dimensionality reduction techniques. These clusters were identified as representing 11 unique cell types, showing considerable diversity among various cell populations (Figure 1A,B). When compared to healthy samples, a significant increase in the proportion of granulocyte subgroups was observed in SCI samples (Figure 1C). Additionally, an in-depth examination of the granulocyte subpopulations identified 16 cell clusters, among which 5 clusters (cluster 0, cluster 1, cluster 2, cluster 3, and cluster 14) exhibited significantly elevated proportions in SCI samples relative to healthy controls, highlighting their potential as subjects for further studies in the context of SCI (Figure 1D,E).

### 3.2. HDWGCNA Algorithm Identifies Key Modules Related to Granulocyte Subgroups

**Figure 2:**
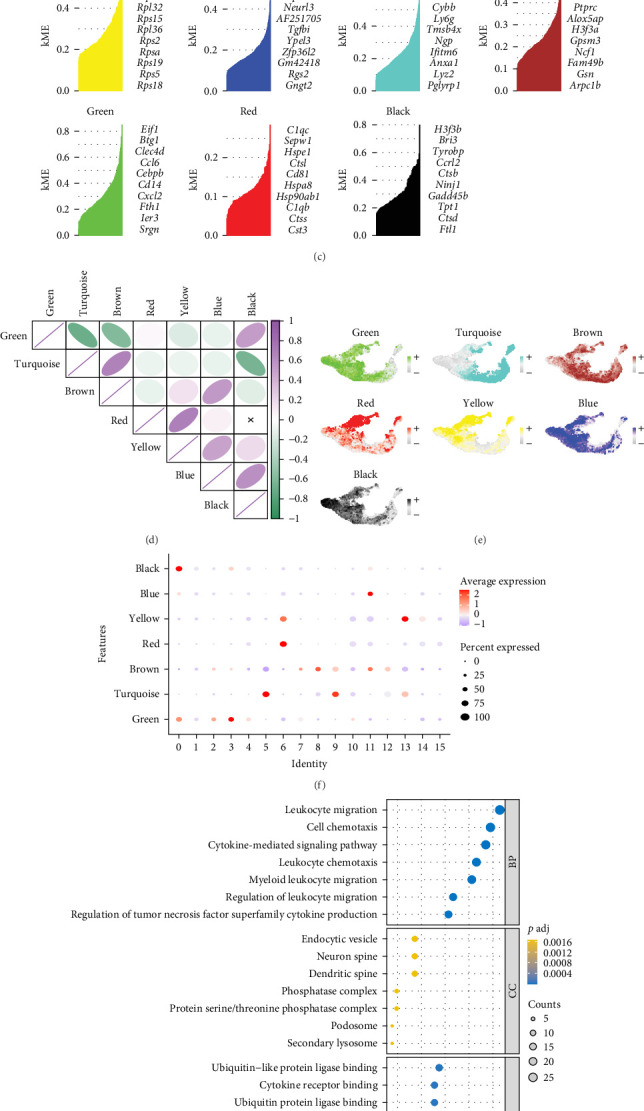
The hdWGCNA illustrates that three central modules, black, green, and yellow, are closely linked to granulocyte subgroups in SCI. (A) A soft power value of 9 was chosen for constructing a scale-free network. (B) To visualize the eight modules in the scale-free network, a tree diagram was utilized. (C) The hdWGCNA pipeline identified eight gene modules, highlighting the top genes. (D) Correlation analysis was conducted among various gene modules. (E) The distribution of the eight gene modules across different granulocyte subgroups is shown. (F) A correlation bubble plot demonstrating the connections between modules and granulocyte subgroups is presented. (G) GO analysis. (H) KEGG analysis.

The hdWGCNA algorithm was utilized to pinpoint crucial modules linked to the molecular characteristics of granulocytes. A scale-free network of fibroblasts was developed based on optimal connectivity, employing a soft threshold value of 9, which resulted in the identification of 8 gene modules in total (Figure 2A–D). The genes in the module are listed in Table S1. We conducted correlation analyses among these 8 gene modules as well as between the modules and granulocyte subgroups. The results demonstrated strong correlations with cluster 0, cluster 1, cluster 2, cluster 3, and cluster 14 (Figure 2E,F). Remarkably, cluster 0 showed a significant correlation with both the black and green modules, while cluster 2 was associated with the green module, cluster 3 was also associated with the green module, and cluster 14 was linked to the yellow module. As a result, we selected the top 100 genes from the black, green, and yellow modules as hub genes for subsequent analysis. The results of GO analysis showed that these genes are significantly correlated with biological functions, such as cytokine-mediated signaling pathways, cell chemotaxis, and leukocyte migration. (Figure 2G) KEGG analysis showed that these genes are associated with biological pathways, such as IL-17 signaling pathway, NF-κ B signaling pathway, JAK/STAT signaling pathway, etc. Significant correlation (Figure 2H).

### 3.3. Screening and WGCNA Analysis of DEGs in SCI Queue

**Figure 3:**
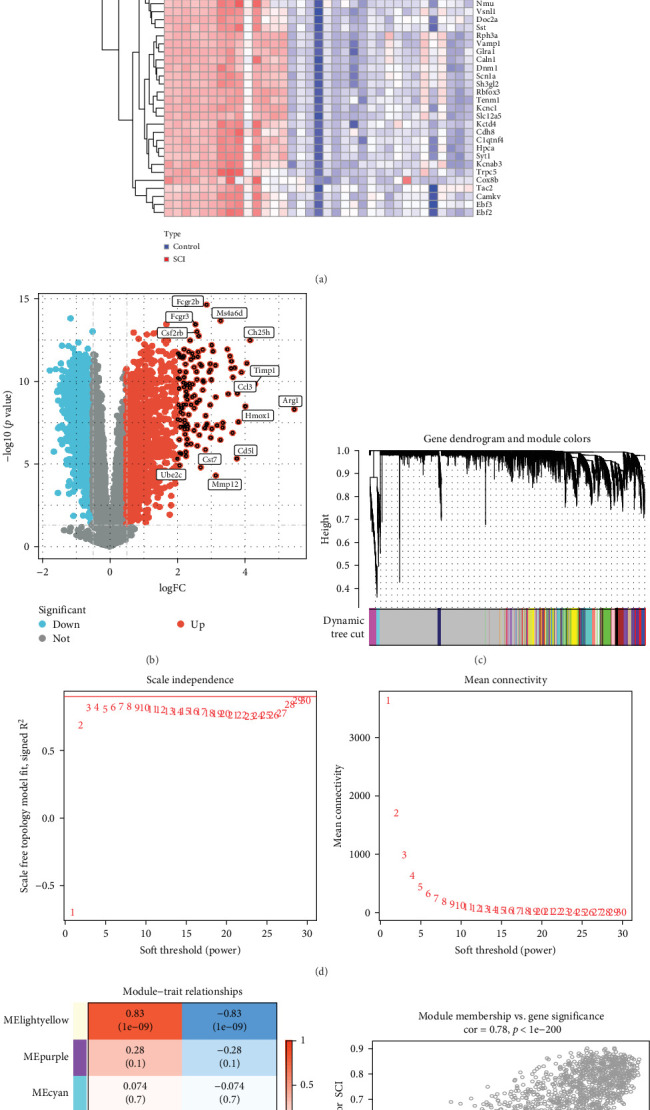
Identification of DEGs associated with SCI and key modules via WGCNA. (A) Heatmap depiction of DEGs. (B) Volcano plot presenting DEGs. (C) Average connectivity and soft threshold power within the WGCNA framework. (D) Dendrogram from the WGCNA analysis. (E) Heat map showing the relationship between modules and clinical features. (F) Dot plot of the relationship between GS and MM for the Gray60 module.

In our investigation of SCI, we detected 751 unique DEGs when comparing SCI samples to normal controls, which included 577 genes that were upregulated and 184 genes that were downregulated (Figure 3A,B). This study also utilized WGCNA to effectively pinpoint crucial genes tied to the SCI phenotype. A soft threshold of 29 was established to maintain the network's scale-free topology (Figure 3C). By analyzing gene correlations, we developed a hierarchical clustering tree diagram of the genes, which allowed for the identification of seven distinct gene modules that displayed similar patterns of co-expression (Figure 3D). To assess the modules that closely relate to SCI, we performed a correlation analysis between each module and the phenotype. Our findings indicated that the “grey60” module showed the strongest clinical association with SCI, comprising 1428 genes (Figure 3E). Moreover, within the “grey60” module, we found a strong relationship between GS and MM, with a correlation coefficient of 0.78 and *p* < 1e − 200 (Figure 3F). As a result, the “grey60” module, along with its core genes, will be included in our subsequent analyses.

### 3.4. Machine Learning Algorithms for Identifying SCI-Related Biomarkers

**Figure 4:**
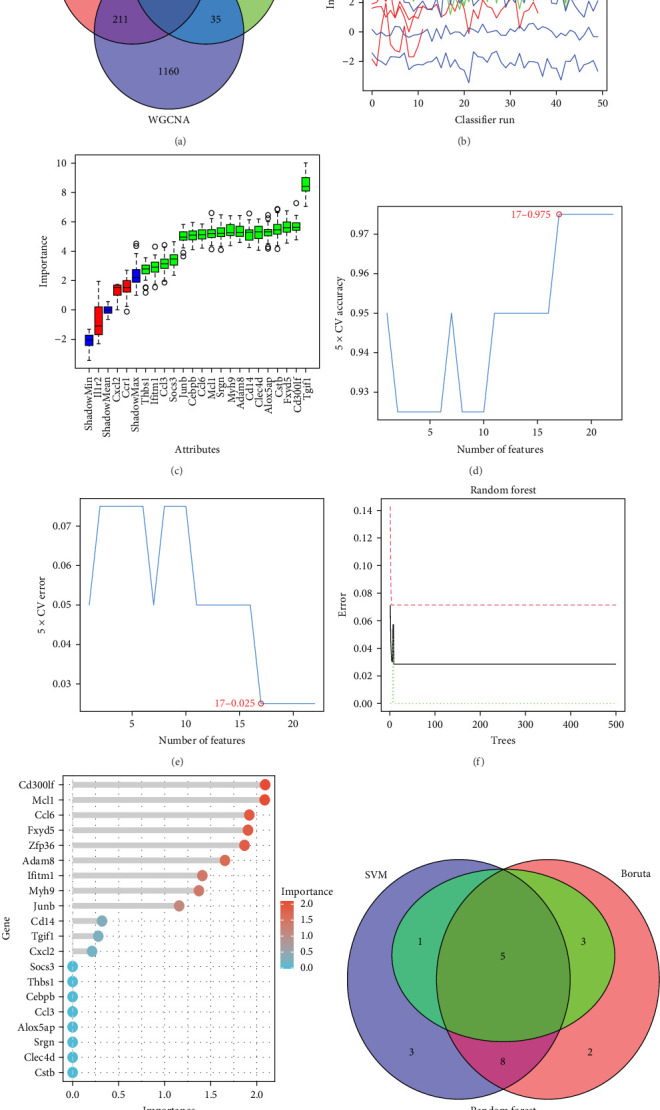
Discovery of biomarkers associated with SCI. (A) The Venn diagram depicts the overlap of genes among the hub genes, DEGs, and core genes of the grey60 module as determined through hdWGCNA. (B, C) Outcomes derived from the Boruta algorithm. (D, E) Results obtained from the SVM-REF algorithm. (F, G) Findings produced by the random forest algorithm. (H) The Venn diagram displays the convergence of biomarkers identified through the three algorithms.

We conducted an intersection analysis of the hub genes, DEGs, and core genes of the grey60 module in hdWGCNA, resulting in the identification of 22 intersecting genes (Figure 4A). Utilizing these 22 intersecting genes, we employed the Boruta algorithm, SVM-RFE, and random forest algorithms to identify biomarkers related to SCI. The Boruta algorithm successfully identified 18 significant biomarkers from the 22 intersecting genes (Figure 4B,C). Subsequently, we screened 17 important biomarkers using the SVM-RFE algorithm (Figure 4D,E). Additionally, the random forest algorithm identified 9 significant biomarkers (Figure 4F,G). Finally, we performed an intersection analysis of the important biomarkers identified by the three algorithms, leading to the identification of five potential biomarkers (Figure 4H).

### 3.5. Identification and Evaluation of Important Biomarkers

**Figure 5:**
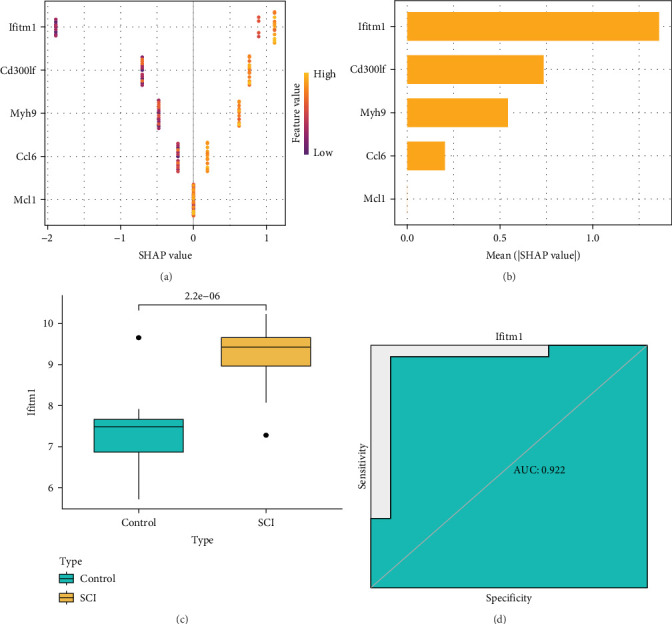
Identification and evaluation of important biomarkers. (A, B) The importance matrix and SHAP summary graph show the genes that contribute to the XGBoost model. (C) There is a significant difference in the expression of Ifitm1 between control samples and SCI samples. (D) ROC evaluation of the clinical diagnostic accuracy of Ifitm1.

We utilized the XGBoost model to assess the significance of five genes, with the goal of identifying feature genes that play a vital role in the model's performance. Following this, we applied SHAP values to clarify the impact of each gene on the model's outcomes. Our findings indicated that Ifitm1 has the highest SHAP value. In addition, we performed SHAP dependency analysis to show how a single feature variable affects the results; notably, a greater SHAP value for this feature variable relates to a higher probability of SCI (Figure 5A,B). As a result, we identified Ifitm1 as the principal gene that has the most substantial influence on model predictions. We further investigated the expression levels of Ifitm1 in control samples versus those in the SCI cohort. The data revealed that the expression of Ifitm1 in SCI samples was significantly elevated compared to control samples (Figure 5C). Moreover, we evaluated the diagnostic accuracy of Ifitm1 clinically through the ROC curve analysis. The findings indicated that the area under the curve (AUC) for Ifitm1 is 0.939 (Figure 5D).

### 3.6. The Relationship Between Ifitm1 and Immune Cells

**Figure 6:**
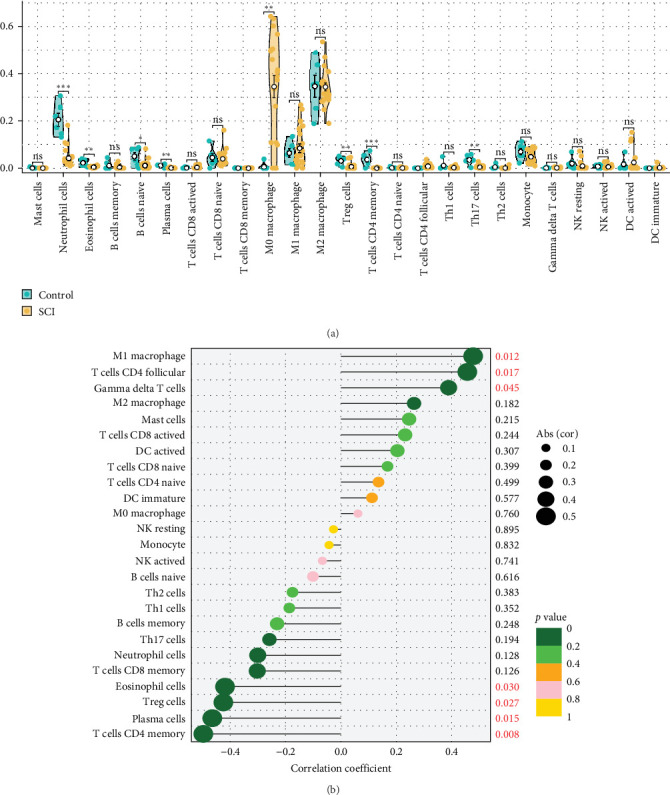
Immune infiltration analysis. (A) Changes in immune cell infiltration levels between SCI samples and normal samples. (B) The correlation between Ifitm1 and immune cells. *⁣*^*∗*^*p* < 0.05; *⁣*^*∗∗*^*p* < 0.01; *⁣*^*∗∗∗*^*p* < 0.001; ns: *p* > 0.05.

To further investigate the alterations in immune cell populations during SCI development, we utilized the CIBERSORT algorithm to assess the immune cell proportions in each sample from the SCI cohort. Our findings demonstrated a notable increase in macrophage M0 levels in SCI samples when compared to those from normal controls. Conversely, the expression levels of neutrophil cells, eosinophil cells, naïve B cells, plasma cells, regulatory T (Treg) cells, CD4 memory T cells, and Th17 cells were significantly heightened in the normal samples (Figure 6A). Additionally, correlation analysis showed a significant positive association between Ifitm1 and macrophage M1, CD4 follicular T cells, and gamma delta T cells, whereas it exhibited a significant negative correlation with CD4 memory T cells, plasma cells, Treg cells, and eosinophil cells (Figure 6B).

### 3.7. GSEA Analysis of Ifitm1

**Figure 7:**
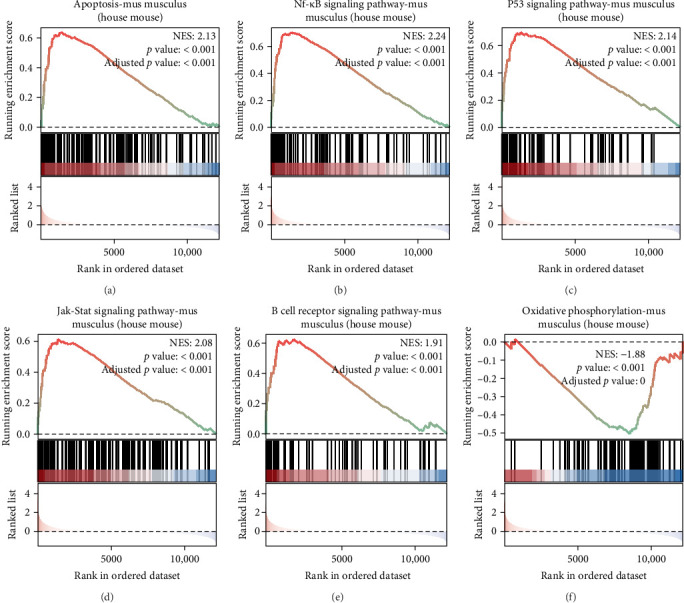
GSEA analysis of Ifitm1. (A) Apoptosis. (B) NF-kappa B signaling pathway. (C) p53 signaling pathway. (D) JAK-STAT signaling pathway. (E) B cell receptor signaling pathway. (F) Oxidative phosphorylation.

To clarify the mechanism through which Ifitm1 operates in SCI, we conducted a Gene Set Enrichment Analysis. The findings revealed a significant association of Ifitm1 with multiple biological pathways, such as apoptosis, the NF-kappa B signaling pathway, the P53 signaling pathway, the JAK-STAT signaling pathway, the B cell receptor signaling pathway, and oxidative phosphorylation (Figure 7).

### 3.8. Drug Prediction and Molecular Docking

**Figure 8:**
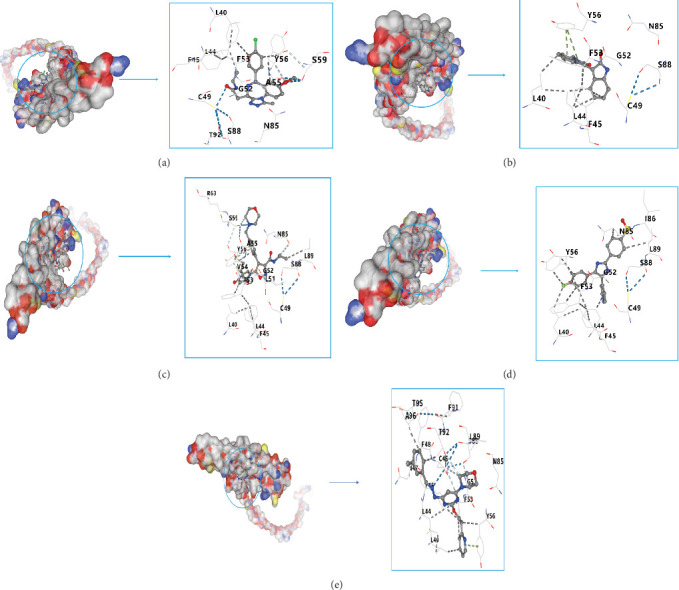
Depicts the binding sites of ligands in proteins. (A) I-BET-762. (B) Meisoindigo. (C) NVP-AUY922. (D) SB-203580. (E) STA-5326.

To predict small-molecule targeted therapies for individuals with SCI, we conducted a differential expression analysis to compare high-risk and low-risk groups within the SCI cohort. We submitted 102 upregulated DEGs to the CMap database, which led to the identification of five targeted drugs based on their connectivity scores. To investigate the most effective small-molecule drugs that interact with the Ifitm1 protein, we performed a molecular docking analysis involving the Ifitm1 protein and the five drugs selected. Our findings from the molecular docking analysis revealed that all examined small-molecule drug compounds exhibited binding energies below −6 kcal/mol, implying favorable interactions with the Ifitm1 target protein. Among these compounds, I-BET-762 had a binding energy of −6.8 kcal/mol, meisoindigo displayed −6.6 kcal/mol, NVP-AUY922 recorded −7.6 kcal/mol, SB-203580 was at −7.4 kcal/mol, and STA-5326 was measured at −7.5 kcal/mol (Figure 8A–E).

### 3.9. Ifitm1 Is Associated With Macrophage Inflammation After SCI

**Figure 9:**
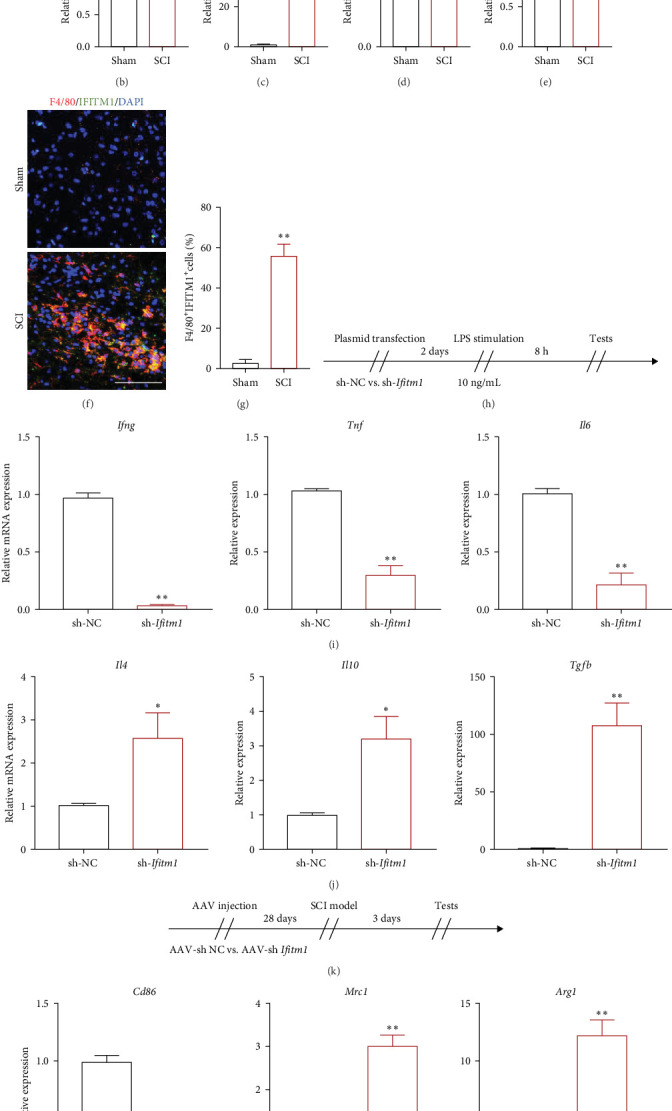
Ifitm1 promotes macrophage inflammation after SCI. (A) FACS strategy for sorting various populations of cells in the spinal cord. (B–E) The expression of Ifitm1 was detected in the cells of each population in the Sham and SCI groups. (F, G) Macrophage colabeling with IFITM1 was detected in the Sham and SCI groups. (H–J) LPS-induced inflammatory changes in macrophages after in vitro inhibition of Ifitm1 expression in macrophages. (K–M) Macrophage phenotype and inflammatory changes after SCI modeling following in vivo inhibition of Ifitm1 expression. (N) Behavioral changes after SCI modeling following in vivo inhibition of Ifitm1 expression. *N* = 3/group. *⁣*^*∗*^*p* ≤ 0.05, *⁣*^*∗∗*^*p* ≤ 0.01. The results are presented as mean ± SEM.

To gain insight into the role of Ifitm1 in the pathology of SCI, we used FACS to sort cell types infiltrating after SCI (Figure 9A). The results of qPCR indicated that Ifitm1 expression was elevated in microglia and macrophages after SCI, whereas it was little altered in lymphocytes and nonimmune cells. Among microglia and macrophages, the highest expression was found in macrophages (Figure 9B–E).

We then examined the co-localization of IFITM1 with macrophages in the spinal cord of mice in the SCI and Sham groups. We could observe a large number of macrophages infiltrating into the spinal cord after injury and high expression of IFITM1 (Figure 9F,G).

To further understand the role of Ifitm1 for macrophages, we interfered with Ifitm1 expression in macrophage RAW264.7 using plasmids in vitro. After silencing Ifitm1 within macrophages, we stimulated macrophages using LPS and detected the expression levels of inflammatory factors by PCR. After silencing Ifitm1 in macrophages, we observed that the transcription of proinflammatory cytokines Ifng, Tnf, and Il6 was downregulated; whereas the transcription of inflammation-suppressing cytokines Il4, Il10, and Tgfb was significantly upregulated. The above results suggest that Ifitm1 is associated with macrophage inflammation after SCI (Figure 9).

We then used AAV, injected into mice via tail vein, to silence systemic Ifitm1 expression. After SCI, macrophages from mice that silenced Ifitm1 exhibited higher M2-like macrophage markers (Mrc1 and Arg1) while Cd86 expression was decreased. Proinflammatory cytokines (Ifng and Tnf) decreased, and inflammation-suppressive cytokines (Tgfb) increased (Figure 9). The quiescence of the inflammatory response aided the recovery of neurological function in sh-Ifitm1 mice (Figure 9N).

## 4. Discussion

SCI is a critical and incapacitating condition that often leads to enduring functional limitations beneath the affected area of the spinal cord. Although there have been notable improvements in the treatment of SCI in recent decades, numerous patients still face prolonged disabilities, resulting in serious repercussions and considerable financial strains on their health [46]. The inappropriate activation of the immune inflammatory response can negatively influence recovery from SCI [47, 48]. The infiltration of immune cells is crucial in the advancement and evolution of SCI [19]. Hence, exploring the immune microenvironment in SCI and pinpointing potential biomarkers for therapeutic approaches in SCI patients is a practical strategy. In this research, we integrated single-cell and microarray datasets to investigate the variations in immune cell infiltration during the course of SCI. In the end, we also recognized biomarkers associated with SCI through the application of machine learning algorithms.

In this study, we analyzed the single-cell dataset GSE189070 and identified significant changes in the granulocyte subpopulation between normal and SCI samples. Granulocytes, a subpopulation of white blood cells, are characterized by multilobular nuclei and cytoplasmic granules. These cells can be distinguished from one another through cytological staining, which allows for the identification of basophils, eosinophils, and neutrophils [49]. In the event of an acute SCI, granulocyte cells migrate into the impacted region, with neutrophils being the initial responders to the injury site and penetrating the lesion. These neutrophils have the ability to produce reactive oxygen species in addition to cytokines and chemokines, which trigger an early inflammatory reaction within the spinal cord [49, 50]. In numerous investigations, neutrophils are viewed as harmful. These cells gather within the inflammatory center of tissue damage, releasing proteases, oxidases, and enzymes that break down tissues, which in turn fosters a damaging tissue environment [49]. Earlier studies have shown that blocking the NF-κB kinase subunit beta can counteract the release of CXCL1 and the ensuing infiltration of neutrophils, while also reducing the expression of proinflammatory genes, ultimately enhancing tissue preservation and mobility [51]. In contrast, the reduction of Ly6G/Gr-1 neutrophils negatively affects the functionality of mice with SCI by obstructing the early recruitment of blood vessels, rolling, and adhesion to endothelial cells, in addition to infiltration into spinal cord tissue. This reduction aggravates the levels of CXCL1, CCL2, G-CSF, and CCL9 in the spinal cord [52]. The involvement of neutrophils is crucial for an appropriate inflammatory response and subsequent tissue repair after SCI. Therefore, understanding the mechanisms that granulocytes utilize during SCI could be an essential treatment strategy. In this study, we utilized the hdWGCNA method to discover gene modules linked to granulocyte subpopulations and identified key genes associated with the onset of SCI. Following this, we characterized the functions of these hub genes. Our findings revealed potential links between these hub genes and various immune responses, as well as inflammatory signals, which include leukocyte migration, interactions between cytokines and their receptors, the IL-17 signaling pathway, NF-κB signaling, the JAK/STAT signaling pathway, and other biological pathways. Importantly, IL-17, a key proinflammatory cytokine mainly produced by Th17 cells, is instrumental in facilitating the inflammatory response [53]. Zong et al. [54] and colleagues discovered that IL-17 could be an important factor in facilitating spinal neuropathy after SCI through the activation of STAT3. As SCI advances, nerve cells and microglia commonly activate NF-*κ*B, leading to the release of a significant quantity of inflammatory cytokines such as IL-1, TNF-α, and IL-6, which in turn worsen the secondary damage that occurs post-SCI [55, 56]. The study conducted by Fei et al. [57] and colleagues indicates that the increased expression of miR-182 may diminish apoptosis in cells and reduce inflammation within spinal cord tissues by blocking the IKK β/NF-κB signaling pathway. Furthermore, the JAK-STAT signaling pathway serves as a vital mediator in how cells respond to inflammation and is crucial for the promotion of carcinogenic processes. This pathway is implicated in a variety of key biological functions, such as cell growth, differentiation, programed cell death, and the regulation of the immune response. Prior research has demonstrated that the JAK/STAT pathway can be triggered following SCI, influencing the proliferation of astrocytes and playing a role in the neuroinflammatory response linked to microglial activation [58–60]. In addition, researchers, including Ma et al. [61] have discovered that Tofacitinib can influence the polarization of microglia and promote functional recovery after SCI via the JAK/STAT pathway.

We combined three datasets derived from mouse models of SCI to investigate potential biomarkers related to SCI. Following this, we utilized three machine learning techniques, Boruta, SVM-RFE, and random forest, to pinpoint Ifitm1 as a biomarker associated with SCI. The protein IFITM1 is implicated in the transduction of homologous adhesion and antiproliferative signals within lymphocytes [62, 63]. In addition, IFITM1 is involved in the physiological processes surrounding the development of primitive germ cells [64, 65]. Prior research has shown that IFITM1 is present in a variety of tissues and cell types, with its levels significantly increasing in response to stimulation by interferons (IFNs) and certain acute-phase cytokines [66, 67]. The research carried out by Wang et al. [68] and colleagues demonstrated that normal spinal cord astrocytes and oligodendrocytes primarily display high levels of IFITM1 expression. Following SCI, a swift increase is noted in infiltrating white blood cells, activated microglia, and astrocytes. Peripheral neutrophils are rapidly attracted to the injury site after SCI, gaining access to the CNS via the damaged blood–brain barrier. Additionally, the release of matrix metalloproteinases (MMPs) aids this infiltration, jeopardizing the neurovascular system's integrity, augmenting neutrophil activity, and thereby facilitating the transendothelial migration of monocytes [69]. When neutrophils experience apoptosis, chemotherapy signals like MCP-1 subsequently attract monocytes to the affected area [17]. Upon arriving at the injury site, these monocytes transform into macrophages due to the influence of various cytokines and chemokines found in the damaged environment, thereby beginning the process of phagocytosing the dying neutrophils [70, 71]. During the initial phase of SCI, macrophages are mainly of the M1 phenotype, aiding in the removal of cellular debris and pathogens; nonetheless, this reaction can also lead to additional tissue harm. This finding is consistent with our analysis, which shows that Ifitm1 is expressed at high levels in SCI and has a notable positive relationship with M1 macrophages. Additionally, macrophages play a complex role in a range of diseases. Unlike M1 macrophages, M2 macrophages are crucial for healing after SCI, as they secrete anti-inflammatory cytokines and neurotrophic factors that foster axonal and nerve repair [72]. The shift of macrophages from M1 to M2 phenotype is crucial for containing injuries and aiding in tissue restoration. A successful response to injury requires precise spatial and temporal control of M1 and M2 macrophages, essential for efficiently clearing the injury area during early phases while encouraging later repair and regeneration. Therefore, strategically managing the balance between M1 and M2 macrophages could act as a therapeutic approach to improve recovery from SCI. Related studies: Ifitm1 expression is associated with immune activation in CD4 T cells, and in SCI, T cell activation and immune responses play a crucial role in the repair and recovery of SCI. Studies have shown that CD4 T cells can modulate the immune response by secreting cytokines, thus affecting the chronic inflammatory response and neural repair in SCI [73]. Meanwhile, a study showed that Th17 cells play an important role in the immune response, especially in the fight against bacterial and fungal infections, by secreting proinflammatory factors such as IL-17. In IVDH, lower levels of Th17 cells and higher levels of IL-17 were detected in dogs during the acute phase [74], suggesting that activation of Th17 cells may lead to excessive inflammatory responses. IL-17 activates neutrophils and promotes their recruitment to the site of injury [75], exacerbating local inflammation. In addition, the balance between Th17 and Treg cells is crucial for the immune response. Treg cells inhibit the overactivation of Th17 cells by secreting anti-inflammatory factors (e.g., IL-10 and TGF-β), preventing uncontrolled immune responses. In IVDH, dysregulation of the balance between Th17 and Treg cells may promote over-activation of proinflammatory Th17 cells, which exacerbates inflammation and facilitates the onset and progression of degenerative disc changes [76]. These provide important research ideas for immunotherapy and repair of human SCI, especially in terms of immune cell regulation, inflammation control, and nerve repair.

Our study delivered new molecular understanding about SCI, but it does have several presentational restrictions. Single-cell RNA sequencing produces detailed cellular data with high resolution, but it comes together with specific operational constraints. Single-cell data resolution reaches its limitations when analyzing significant numbers of cells and covered genes, which might miss detecting low-abundance gene expressions. The classification and annotation methods for specific cell populations remain challenging through single-cell analysis, particularly when tissue environments are complex, as some cell clusters obtain inaccurate identification. Furthermore, the study examined Ifitm1-M1 macrophage relationships. Although the SCI immune response requires investigation of numerous immune cell types interacting with one another. Many immune system cells work together to defend against spinal cord injuries through a mechanism that includes dendritic cells along with T cells and several others. Future studies should extend their research to analyze various immune cell functions and interactions in order to fully understand how the immune system responds after a SCI.

## 5. Conclusion

In conclusion, we recognized granulocyte subpopulations as separate entities in SCI utilizing single-cell analysis. Following this, we combined this information with a mouse microarray dataset and applied machine learning algorithms to pinpoint Ifitm1 as a central gene in SCI. Importantly, Ifitm1 showed a notable positive association with M1 macrophages. Experimental findings further illustrated that Ifitm1 is linked to macrophage inflammation after SCI. Overall, the results of this study offer new insights into the molecular mechanisms involved in SCI and propose potential therapeutic targets.

## Figures and Tables

**Table 1:** The sequence of si-Ifitm1.

Gene	Target sequences (5′-3′)
si-*Ifitm1*	AGGATTCCAAGCAAAGATCTTCC

**Table 2:** The primer sequence of qRT-PCR.

Gene	Forward primer sequence (5′-3′)	Reverse primer sequence (5′-3′)
*Ifitm1*	GCCACCACAATCAACATGCCTG	ACCCACCATCTTCCTGTCCCTA
*Ifng*	CAGCAACAGCAAGGCGAAAAAGG	TTTCCGCTTCCTGAGGCTGGAT
*Tnf*	GGTGCCTATGTCTCAGCCTCTT	GCCATAGAACTGATGAGAGGGAG
*Il6*	TACCACTTCACAAGTCGGAGGC	CTGCAAGTGCATCATCGTTGTTC
*Il4*	ATCATCGGCATTTTGAACGAGGTC	ACCTTGGAAGCCCTACAGACGA
*Il10*	CGGGAAGACAATAACTGCACCC	CGGTTAGCAGTATGTTGTCCAGC
*Tgfb1*	TGATACGCCTGAGTGGCTGTCT	CACAAGAGCAGTGAGCGCTGAA
*Cd86*	ACGTATTGGAAGGAGATTACAGCT	TCTGTCAGCGTTACTATCCCGC
*Mrc1*	GTTCACCTGGAGTGATGGTTCTC	AGGACATGCCAGGGTCACCTTT
*Arg1*	CATTGGCTTGCGAGACGTAGAC	GCTGAAGGTCTCTTCCATCACC
*Actb*	CATTGCTGACAGGATGCAGAAGG	TGCTGGAAGGTGGACAGTGAGG

## Data Availability

The datasets analyzed in the current study (GSE47681, GSE92657, GSE93561, and GSE189070) are available in the GEO repository (https://www.ncbi.nlm.nih.gov/geo/).

## Supporting Information

Supporting Information Table S1. All genes in the eight modules.

## References

[1] Wang H., Zhou W.-X., Huang J.-F. (2020). Endocrine Therapy for the Functional Recovery of Spinal Cord Injury. *Frontiers in Neuroscience*.

[2] Khorasanizadeh M. H., Yousefifard M., Eskian M. (2019). Neurological Recovery Following Traumatic Spinal Cord Injury: A Systematic Review and Meta-Analysis. *Journal of Neurosurgery: Spine*.

[3] Keihanian F., Kouchakinejad-Eramsadati L., Yousefzadeh-Chabok S. (2022). Burden in Caregivers of Spinal Cord Injury Patients: A Systematic Review and Meta-Analysis. *Acta Neurologica Belgica*.

[4] Ahuja C. S., Nori S., Tetreault L. (2017). Traumatic Spinal Cord Injury—Repair and Regeneration. *Neurosurgery*.

[5] Ahuja C. S., Wilson J. R., Nori S. (2017). Traumatic Spinal Cord Injury. *Nature Reviews Disease Primers*.

[6] Hewson D. W., Bedforth N. M., Hardman J. G. (2018). Spinal Cord Injury Arising in Anaesthesia Practice. *Anaesthesia*.

[7] Ge L., Arul K., Mesfin A. (2019). Spinal Cord Injury From Spinal Tumors: Prevalence, Management, and Outcomes. *World Neurosurgery*.

[8] Krause J. S., Cao Y., DeVivo M. J., DiPiro N. D. (2016). Risk and Protective Factors for Cause-Specific Mortality After Spinal Cord Injury. *Archives of Physical Medicine and Rehabilitation*.

[9] Fakhoury M. (2015). Spinal Cord Injury: Overview of Experimental Approaches Used to Restore Locomotor Activity. *Reviews in the Neurosciences*.

[10] Cao L., Li Q. (2022). Revealing Potential Spinal Cord Injury Biomarkers and Immune Cell Infiltration Characteristics in Mice. *Frontiers in Genetics*.

[11] Samantaray S., Das A., Matzelle D. C. (2016). Administration of Low Dose Estrogen Attenuates Persistent Inflammation, Promotes Angiogenesis, and Improves Locomotor Function Following Chronic Spinal Cord Injury in Rats. *Journal of Neurochemistry*.

[12] Carelli S., Giallongo T., Rey F. (2019). Recovery of Function and Endogenous Neurogenesis in Traumatic Spinal Cord Injury Following Transplantation of Activated Adipose Tissue. *Cells*.

[13] Saremi J., Mahmoodi N., Rasouli M. (2022). Advanced Approaches to Regenerate Spinal Cord Injury: The Development of Cell and Tissue Engineering Therapy and Combinational Treatments. *Biomedicine & Pharmacotherapy*.

[14] Alizadeh A., Dyck S. M., Karimi-Abdolrezaee S. (2019). Traumatic Spinal Cord Injury: An Overview of Pathophysiology, Models and Acute Injury Mechanisms. *Frontiers in Neurology*.

[15] Wang P., Qi X., Xu G. (2019). CCL28 Promotes Locomotor Recovery After Spinal Cord Injury via Recruiting Regulatory T Cells. *Aging*.

[16] Xu P., Zhang F., Chang M.-M. (2021). Recruitment of *γ*δ T Cells to the Lesion via the CCL2/CCR2 Signaling After Spinal Cord Injury. *Journal of Neuroinflammation*.

[17] Milich L. M., Ryan C. B., Lee J. K. (2019). The Origin, Fate, and Contribution of Macrophages to Spinal Cord Injury Pathology. *Acta Neuropathologica*.

[18] Ahmed A., Patil A.-A., Agrawal D. K. (2018). Immunobiology of Spinal Cord Injuries and Potential Therapeutic Approaches. *Molecular and Cellular Biochemistry*.

[19] Al Mamun A., Monalisa I., Tul Kubra K. (2021). Advances in Immunotherapy for the Treatment of Spinal Cord Injury. *Immunobiology*.

[20] Qiong L., Shuyao X., Shan X. (2024). Recent Advances in the Glycolytic Processes Linked to Tumor Metastasis. *Current Molecular Pharmacology*.

[21] Lim D. V., Woo W. H., Lim J. X. (2023). Targeting Mutant-p53 for Cancer Treatment: Are We There Yet?. *Current Molecular Pharmacology*.

[22] Liu L., Xie Y., Yang H. (2023). HPVTIMER: A Shiny Web Application for Tumor Immune Estimation in Human Papillomavirus-Associated Cancers. *iMeta*.

[23] Zhang W., Xie X., Huang Z. (2022). The Integration of Single-Cell Sequencing, TCGA, and GEO Data Analysis Revealed That PRRT3-AS1 Is a Biomarker and Therapeutic Target of SKCM. *Frontiers in Immunology*.

[24] Yu L., Shen N., Shi Y. (2022). Characterization of Cancer-Related Fibroblasts (CAF) in Hepatocellular Carcinoma and Construction of CAF-Based Risk Signature Based on Single-Cell RNA-Seq and Bulk RNA-Seq Data. *Frontiers in Immunology*.

[25] Yu X., Xie L., Ge J., Li H., Zhong S., Liu X. (2023). Integrating Single-Cell RNA-Seq and Spatial Transcriptomics Reveals MDK-NCL Dependent Immunosuppressive Environment in Endometrial Carcinoma. *Frontiers in Immunology*.

[26] Huang R., Wang W., Chen Z. (2023). Identifying Immune Cell Infiltration and Effective Diagnostic Biomarkers in Crohn’s Disease by Bioinformatics Analysis. *Frontiers in Immunology*.

[27] Zhang T., Wan L., Xiao H., Wang L., Hu J., Lu H. (2023). Single-Cell RNA Sequencing Reveals Cellular and Molecular Heterogeneity in Fibrocartilaginous Enthesis Formation. *eLife*.

[28] Yan Q., Zhao Z., Liu D. (2024). Novel Immune Cross-Talk Between Inflammatory Bowel Disease and IgA Nephropathy. *Renal Failure*.

[29] Xu M., Zhou H., Hu P. (2023). Identification and Validation of Immune and Oxidative Stress-Related Diagnostic Markers for Diabetic Nephropathy by WGCNA and Machine Learning. *Frontiers in Immunology*.

[30] Zhu E., Shu X., Xu Z. (2023). Screening of Immune-Related Secretory Proteins Linking Chronic Kidney Disease With Calcific Aortic Valve Disease Based on Comprehensive Bioinformatics Analysis and Machine Learning. *Journal of Translational Medicine*.

[31] Liu F., Huang Y., Liu F., Wang H. (2023). Identification of Immune-Related Genes in Diagnosing Atherosclerosis With Rheumatoid Arthritis Through Bioinformatics Analysis and Machine Learning. *Frontiers in Immunology*.

[32] Lin A., Jiang A., Huang L. (2025). From Chaos to Order: Optimizing Fecal Microbiota Transplantation for Enhanced Immune Checkpoint Inhibitors Efficacy. *Gut Microbes*.

[33] Wang Z., Zhao Y., Zhang L. (2024). Emerging Trends and Hot Topics in the Application of Multi-Omics in Drug Discovery: A Bibliometric and Visualized Study. *Current Pharmaceutical Analysis*.

[34] Chen J., Lin A., Luo P. (2024). Advancing Pharmaceutical Research: A Comprehensive Review of Cutting-Edge Tools and Technologies. *Current Pharmaceutical Analysis*.

[35] Leek J. T., Johnson W. E., Parker H. S., Jaffe A. E., Storey J. D. (2012). The sva Package for Removing Batch Effects and Other Unwanted Variation in High-Throughput Experiments. *Bioinformatics*.

[36] Leek J. T., Scharpf R. B., Bravo H. C. (2010). Tackling the Widespread and Critical Impact of Batch Effects in High-Throughput Data. *Nature Reviews Genetics*.

[37] Stuart T., Butler A., Hoffman P. (2019). Comprehensive Integration of Single-Cell Data. *Cell*.

[38] Morabito S., Reese F., Rahimzadeh N., Miyoshi E. (2023). hdWGCNA Identifies Co-Expression Networks in High-Dimensional Transcriptomics Data. *Cell Reports Methods*.

[39] Chen Y., Liao R., Yao Y., Wang Q., Fu L. (2022). Machine Learning to Identify Immune-Related Biomarkers of Rheumatoid Arthritis Based on WGCNA Network. *Clinical Rheumatology*.

[40] Kursa M. B. (2014). Robustness of Random Forest-Based Gene Selection Methods. *BMC Bioinformatics*.

[41] Sanz H., Valim C., Vegas E., Oller J. M., Reverter F. (2018). SVM-RFE: Selection and Visualization of the Most Relevant Features Through Non-Linear Kernels. *BMC Bioinformatics*.

[42] Garge N. R., Bobashev G., Eggleston B. (2013). Random Forest Methodology for Model-Based Recursive Partitioning: The mobForest Package for R. *BMC Bioinformatics*.

[43] Newman A. M., Liu C. L., Green M. R. (2015). Robust Enumeration of Cell Subsets From Tissue Expression Profiles. *Nature Methods*.

[44] Liu Y., Grimm M., Dai W.-T., Hou M.-C., Xiao Z.-X., Cao Y. (2020). CB-Dock: A Web Server for Cavity Detection-Guided Protein-Ligand Blind Docking. *Acta Pharmacologica Sinica*.

[45] Cao Y., Li L. (2014). Improved Protein-Ligand Binding Affinity Prediction by Using a Curvature-Dependent Surface-Area Model. *Bioinformatics*.

[46] Wu C., Yu J., Xu G. (2021). Bioinformatic Analysis of the Proteome in Exosomes Derived From Plasma: Exosomes Involved in Cholesterol Metabolism Process of Patients With Spinal Cord Injury in the Acute Phase. *Frontiers in Neuroinformatics*.

[47] Sterner R. C., Sterner R. M. (2023). Immune Response Following Traumatic Spinal Cord Injury: Pathophysiology and Therapies. *Frontiers in Immunology*.

[48] Jin Y., Song Y., Lin J. (2023). Role of Inflammation in Neurological Damage and Regeneration Following Spinal Cord Injury and Its Therapeutic Implications. *Burns & Trauma*.

[49] Neirinckx V., Coste C., Franzen R., Gothot A., Rogister B., Wislet S. (2014). Neutrophil Contribution to Spinal Cord Injury and Repair. *Journal of Neuroinflammation*.

[50] Guo X., Jiang C., Chen Z., Wang X., Hong F., Hao D. (2023). Regulation of the JAK/STAT Signaling Pathway in Spinal Cord Injury: An Updated Review. *Frontiers in Immunology*.

[51] Kang J., Jiang M. H., Min H. J. (2011). IKK-*β*-Mediated Myeloid Cell Activation Exacerbates Inflammation and Inhibits Recovery After Spinal Cord Injury. *European Journal of Immunology*.

[52] Stirling D. P., Liu S., Kubes P., Yong V. W. (2009). Depletion of Ly6G/Gr-1 Leukocytes After Spinal Cord Injury in Mice Alters Wound Healing and Worsens Neurological Outcome. *The Journal of Neuroscience*.

[53] Korn T., Bettelli E., Oukka M., Kuchroo V. K. (2009). IL-17 and Th17 Cells. *Annual Review of Immunology*.

[54] Zong S., Zeng G., Fang Y. (2014). The Role of IL-17 Promotes Spinal Cord Neuroinflammation via Activation of the Transcription Factor STAT3 After Spinal Cord Injury in the Rat. *Mediators of Inflammation*.

[55] Bulek K., Liu C., Swaidani S. (2011). The Inducible Kinase IKKi Is Required for IL-17-Dependent Signaling Associated With Neutrophilia and Pulmonary Inflammation. *Nature Immunology*.

[56] Awane M., Andres P. G., Li D. J., Reinecker H. C. (1999). NF-*κ*B-Inducing Kinase Is a Common Mediator of IL-17-, TNF-*α*-, and IL-1*β*-Induced Chemokine Promoter Activation in Intestinal Epithelial Cells. *The Journal of Immunology*.

[57] Fei M., Li Z., Cao Y., Jiang C., Lin H., Chen Z. (2021). MicroRNA-182 Improves Spinal Cord Injury in Mice by Modulating Apoptosis and the Inflammatory Response via IKK*β*/NF-*κ*B. *Laboratory Investigation*.

[58] Yamauchi K., Osuka K., Takayasu M. (2006). Activation of JAK/STAT Signalling in Neurons Following Spinal Cord Injury in Mice. *Journal of Neurochemistry*.

[59] Tsuda M., Kohro Y., Yano T. (2011). JAK-STAT3 Pathway Regulates Spinal Astrocyte Proliferation and Neuropathic Pain Maintenance in Rats. *Brain*.

[60] Butturini E., Boriero D., Carcereri de Prati A., Mariotto S. (2019). STAT1 Drives M1 Microglia Activation and Neuroinflammation Under Hypoxia. *Archives of Biochemistry and Biophysics*.

[61] Ma H., Wang C., Han L. (2023). Tofacitinib Promotes Functional Recovery After Spinal Cord Injury by Regulating Microglial Polarization via JAK/STAT Signaling Pathway. *International Journal of Biological Sciences*.

[62] Lewin A. R., Reid L. E., McMahon M., Stark G. R., Kerr I. M. (1991). Molecular Analysis of a Human Interferon-Inducible Gene Family. *European Journal of Biochemistry*.

[63] Sato S., Miller A. S., Howard M. C., Tedder T. F. (1997). Regulation of B Lymphocyte Development and Activation by the CD19/CD21/CD81/Leu 13 complex Requires the Cytoplasmic Domain of CD19. *The Journal of Immunology*.

[64] Tanaka S. S., Yamaguchi Y. L., Tsoi B., Lickert H., Tam P. P. (2005). IFITM/Mil/Fragilis Family Proteins IFITM1 and IFITM3 Play Distinct Roles in Mouse Primordial Germ Cell Homing and Repulsion. *Developmental Cell*.

[65] Wylie C. (2005). IFITM1-Mediated Cell Repulsion Controls the Initial Steps of Germ Cell Migration in the Mouse. *Developmental Cell*.

[66] Bailey C. C., Zhong G., Huang I.-C., Farzan M. (2014). IFITM-Family Proteins: The Cell’s First Line of Antiviral Defense. *Annual Review of Virology*.

[67] Diamond M. S., Farzan M. (2013). The Broad-Spectrum Antiviral Functions of IFIT and IFITM Proteins. *Nature Reviews Immunology*.

[68] Wang Y., Lin Y. H., Wu Y. (2018). Expression and Cellular Localization of IFITM1 in Normal and Injured Rat Spinal Cords. *Journal of Histochemistry & Cytochemistry*.

[69] Noble L. J., Donovan F., Igarashi T., Goussev S., Werb Z. (2002). Matrix Metalloproteinases Limit Functional Recovery After Spinal Cord Injury by Modulation of Early Vascular Events. *The Journal of Neuroscience*.

[70] Hawthorne A. L., Popovich P. G. (2011). Emerging Concepts in Myeloid Cell Biology After Spinal Cord Injury. *Neurotherapeutics*.

[71] Kigerl K. A., Gensel J. C., Ankeny D. P., Alexander J. K., Donnelly D. J., Popovich P. G. (2009). Identification of Two Distinct Macrophage Subsets With Divergent Effects Causing Either Neurotoxicity or Regeneration in the Injured Mouse Spinal Cord. *The Journal of Neuroscience*.

[72] Wang J., Tian F., Cao L. (2023). Macrophage Polarization in Spinal Cord Injury Repair and the Possible Role of microRNAs: A Review. *Heliyon*.

[73] Canoui E., Noël N., Lécuroux C. (2017). Strong ifitm1 Expression in CD4 T Cells in HIV Controllers Is Correlated With Immune Activation. *Journal of Acquired Immune Deficiency Syndromes*.

[74] Guéry L., Hugues S. (2015). Th17 Cell Plasticity and Functions in Cancer Immunity.. *BioMed Research International*.

[75] Curtis M. M., Way S. S. (2009). Interleukin-17 in Host Defence Against Bacterial, Mycobacterial and Fungal Pathogens. *Immunology*.

[76] Cheng L., Fan W., Liu B., Wang X., Nie L. (2013). Th17 Lymphocyte Levels Are Higher in Patients With Ruptured than Non-Ruptured Lumbar Discs, and Are Correlated With Pain Intensity. *Injury*.

